# Supplementary material for: Utility of Self-Reported Heat Stress Symptoms and NGAL Biomarker to Screen for Chronic Kidney Disease of Unknown Origin (CKDu) in Sri Lanka
Source: Int J Environ Res Public Health. 2021 Oct 6;18(19):10498. doi: 10.3390/ijerph181910498 (PMC8507708; doi:10.3390/ijerph181910498)
Supplement: Supplementary file 1 [file ijerph-18-10498-s001.zip › ijerph-1370051-supplementary.pdf]

Supplement: NGAL Laboratory Protocol

Four urine samples were collected.

1. Sample A. 25 ml of early-morning first-voided urine sample collected when participants leave for work around 5.00-8.00 am. Sample collection cup with instructions provided the previous day.
2. Sample B. 25 ml of urine 4.00-6.00 pm just after finishing work collected at workplaces. Sample collection cups will be provided for this sample when collecting the morning sample.
3. Sample C. 25 ml of urine in the evening after having few hours rest and after the participant is well rehydrated, 7.00-10.00pm. Sample collection cups will be provided for this sample when collecting the second sample.
4. Sample D. 25 ml of early-morning first-voided urine collected the next day when participants leave for work around 5.00-8.00 am. The sample collection cup will be provided to them in the previous day when collecting the third urine sample.

- Urine samples were centrifuged at 3000 rpm for 10 minutes at stations set in the field directly after sample collection within one hour.
- Supernatant of samples were stored in Eppendorfs and frozen at -80^{0}C.
- Urine NGAL was measured by particle enhance turbidimetric immunoassay method using Roche Cobas C 501 biochemistry analyzer, which is avalable in Lanka Hospital Diagnostic Laboratory. Urine albumin was measured using turbidimetric immunoassay, and urine creatinine was measured by Modified Jaffé method.
- All the urine tests were performed under the supervision of Dr. Saroja Siriwardena, Chemical Pathologist., Lanka Hospital Diagnostics Laboratory.
- Urine SG was measured by Refractometer.
